# *PbUGT72AJ2*-Mediated Glycosylation Plays an Important Role in Lignin Formation and Stone Cell Development in Pears (*Pyrus bretschneideri*)

**DOI:** 10.3390/ijms23147893

**Published:** 2022-07-18

**Authors:** Han Wang, Xiaofeng Feng, Yingjie Zhang, Dongyi Wei, Yang Zhang, Qing Jin, Yongping Cai

**Affiliations:** 1 School of Life Sciences, Anhui Agricultural University, Hefei 230036, China; wh0707@stu.ahau.edu.cn (H.W.); feng1997@stu.ahau.edu.cn (X.F.); yingjie_zhang@stu.ahau.edu.cn (Y.Z.); 337241296@stu.ahau.edu.cn (D.W.); zhangyang@ahau.edu.cn (Y.Z.); qingjin@ahau.edu.cn (Q.J.); 2 Anhui Provincial Engineering Technology Research Center for Development & Utilization of Regional Characteristic Plants, Anhui Agricultural University, No. 130, Changjiang West Road, Hefei 230036, China

**Keywords:** *Pyrus bretschneideri*, lignin biosynthesis, stone cells, glycosylation

## Abstract

Glycosylation is necessary for many processes of plant secondary metabolism. It can maintain plant homeostasis and is of great significance to normal plant growth and development. At present, the significance of glycosylation for lignin biosynthesis has been proven in some plants, but it has not yet been reported in pears. We used in situ hybridization, in vitro expression, substrate catalysis, transgenic *Arabidopsis* *thaliana*, and transient transformation of pear fruit in our investigation, which was predicated on the identification of a gene *PbUGT72AJ2* that may be involved in lignin monolignol glycosylation according to our previous work. These results revealed that *PbUGT72AJ2* transcripts were localized to some pulp cell walls, lignin deposition, and stone cell areas of pear fruit. The recombinant PbUGT72AJ2-pGEX4T-1 protein had activity against coniferyl alcohol and sinapyl alcohol, and its catalytic efficiency against coniferyl alcohol was higher than that against sinapyl alcohol. When *PbUGT72AJ2* was transferred into *Arabidopsis* *thaliana* mutants, it was found that some characteristics of *Arabidopsis* *thaliana* *ugt72e3* mutants were restored. In *Arabidopsis* *thaliana*, overexpression of *PbUGT72AJ2* enhanced the contents of coniferin and syringin, whereas lignification did not change significantly. Transient transformation of pear fruit showed that when *PbUGT72AJ2* in pear fruit was silenced by RNA interference, the content of lignin and stone cells in pear fruit increased, whereas the gene *PbUGT72AJ2* was overexpressed in pear fruit, and there was almost no change in the pear fruit compared with the control. Lignin deposition in pear fruit was closely related to stone cell development. In this study, we proved that *PbUGT72AJ2* plays an important role in lignin deposition and stone cell development in pear fruit, which provides a molecular biological basis for improving pear fruit quality at the molecular level.

## 1. Introduction

Pear belongs to the subtribe *Malinae* of the family *Rosaceae* and is one of the most important deciduous fruit tree species worldwide [1]. Pear fruit is not only delicious, juicy, sweet, and sour but also rich in nutrition. However, pear fruit contains a large number of stone cells, which is an important factor affecting the quality of pear fruit, and *Pyrus bretschneideri* is even more affected than other pear varieties [2,3,4]. Lignin is the main component in pear fruit stone cells. The lignin content in mature pear fruit stone cells is approximately 20–40%, and the stone cell content is positively correlated with the lignin content [5,6]. Previous studies have found that the development of pear fruit stone cells generally includes two important stages: one is the transport and deposition of lignin and the other is the uneven thickening of the secondary wall [7]. During the development of the *Pyrus bretschneideri* stone cells of fruit, a large amount of lignin is deposited on the cell wall of the parenchymal cells, which thickens the cell wall, thereby forming thick-walled cells [8]. Furthermore, the transport of lignin monolignols are key factors affecting the formation of stone cells. Lignin monolignols (coniferyl alcohol, sinapyl alcohol) are synthesized in the cytoplasm, but their polymerization occurs outside the cell membrane [9]. In recent years, studies have found that lignin monolignols undergo glycosylation modification during transportation [10,11]. Therefore, studying glycosyltransferases involved in lignin metabolism in pears is crucial.

Although monolignols are synthesized in cell protoplasts, the deposition of lignin is limited to the cell wall. There is no definite answer to the question of via which transmembrane mode these monolignols are transported outside the membrane. At present, academia believes that there are three transport mechanisms for the extracellular secretion of monolignols, namely passive diffusion (PD), vesicle-related exocytosis and active transport of ATP consumed by ABC transport factor or proton-coupled reverse transporter [12,13,14]. In recent years, through the identification of the chemical composition of each component in the lignin synthesis pathway, it has been found that most monolignols in cells exist in the form of free glycosides (no sugar compounds) or 4-O-B-d-glucosides, namely coniferin, syringin, and *p*-coumarin [15], and this situation is more obvious in gymnosperm [16]. Incomplete sugar binding occurs in some species of angiosperms; for example, coniferin and syringin accumulate in *Arabidopsis* *thaliana* roots, and their contents can be increased by light treatment [17,18]. From a chemical point of view, sugar binding increases stability and water solubility, and monolignol glycosides may exist in vacuoles of different xylem cells [18,19]. Some researchers have also suggested that these monolignol glucosides may be isolated from cell vacuoles and then exported to the cell wall [20,21]. The above research results show that these monolignol glucosides may play a role in the storage and transportation of monolignols. A very important enzyme is required to participate from monolignol to its glycoside form, namely uridine diphosphate glycosyltransferase (UGT). *UGTs* usually use UDP glucose or UDP galactose as sugar donors to dehydrogenate and combine the activated sugar group with a hydroxyl group on monolignol to form the glycoside form of monolignol [22]. After these monolignol glycosides are transported to a specific location on the extracellular cell wall through the three transport mechanisms mentioned above, these monolignol glucosides are then transported to specific parts of the cell wall and hydrolysed to lignin monolignols under the action of β-glucosidase [23,24,25], ultimately forming guaiacyl (G), syringyl (S), and *p*-hydroxyphenyl (H) units by laccase and peroxidase (dicotyledons have only guaiacyl (G) and syringyl (S) lignin) [26,27,28]. In previous studies, it has been found that *ugt72e1–3* of *Arabidopsis thaliana* encode glycosyltransferases that have been shown to glucosylate several phenylpropanoids in vitro, including monolignols, hydroxycinnamic acids, and hydroxycinnamic aldehydes [29,30], and a study found that the glucosyltransferase *ugt72e2* is responsible for monolignol 4-O-glucoside production in *Arabidopsis thaliana* [31,32]. In addition, the identification of a mutant *ugt72b1* showed aggravated and ectopic lignification in floral stems along with arrested growth and anthocyanin accumulation. Histochemical assays and thioacidolysis analysis confirmed the enhanced lignification and increased lignin biosynthesis in the *ugt72b1* mutant [33]. Recent research has found that the use of a novel safranin O ratiometric imaging technique indicated a significant increase in the cell wall lignin content of both interfascicular fibres and xylem from young regions of *ugt72e3* mutant floral stems [34]. 

At present, all members of the glycosyltransferase family in pears have been screened and identified, and we have identified UDP uridine diphosphate glycosyltransferase (UGT) *Pbr005014.1* (named *PbUGT72AJ2* GenBank: KR270486). The gene was considered a functional gene for pear lignin synthesis [25]. Current investigations include in vitro enzyme activity to study the catalytic activity of PbUGT72AJ2 for monolignols and PbUGT72AJ2 enzyme kinetic characteristics in vitro, using in situ hybridization to determine the expression position of *PbUGT72AJ2* in pear fruit; stable transformation of *Arabidopsis thaliana* in vivo, and transient expression in pear fruit revealed that *PbUGT72AJ2* played a vital role in the formation of lignin and stone cell development in pear fruit. This study provides the groundwork for improving the quality of pear fruit at the molecular level.

## 2. Results

### 2.1. Phylogenetic and Sequence Analyses of PbUGT72AJ2

**Figure 1:**
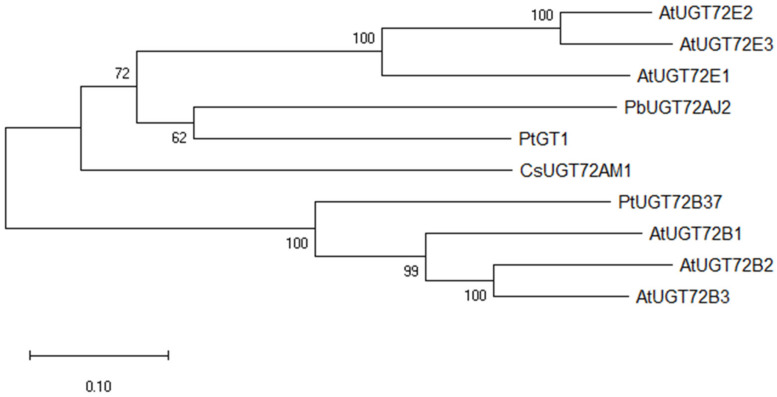
Phylogenetic tree of *PbUGT72AJ2* and *UGT* homologues from other plants. The phylogenetic tree was constructed using MEGA 7.0 [38] with the neighbor-joining method.

**Figure 2:**
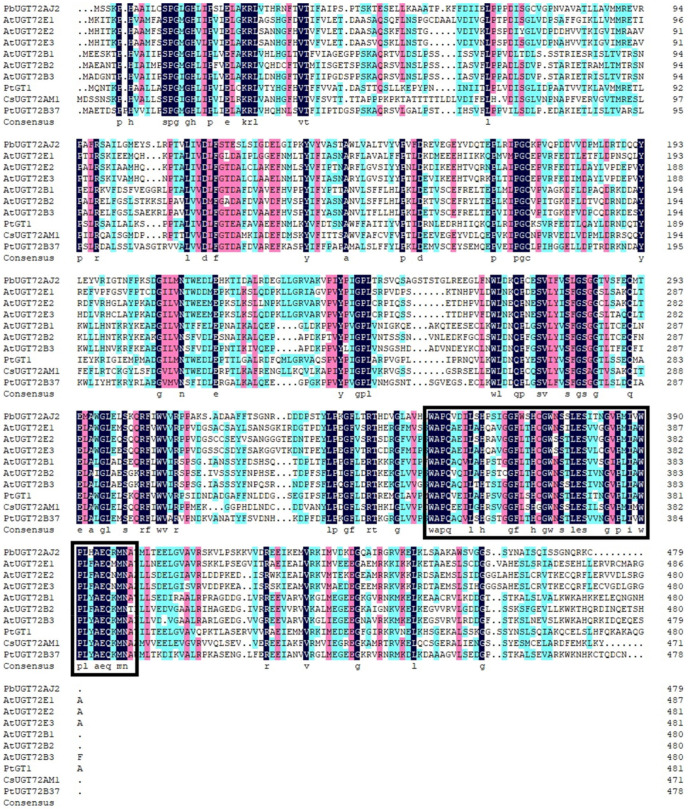
Amino acid sequence alignment of PbUGT72AJ2 and nine homologues. The identified UGTs in the multiple alignment are AtUGT72E1, 2, 3 (*A. thaliana* At3g50740, At5g66690, At5g26310); AtUGT72B1, 2, 3 (*A. thaliana* At4g01070, At1g01390, At1g01420); *Populus tomentosa* PGT1 (GenBank: HM776516), *Camellia sinensis* CsUGT72AM1 (GenBank: KY399734) and *Populus trichocarpa* UGT72B37 (GenBank: MT181030). The multiple alignment was performed using DNAMAN 7.0 software (LynnonBiosoft, San Ramon, CA, USA). The black rectangle indicates the domains of the PSPG box; Different colors indicate different homology.

In a previous study, 139 *UGT*s were identified from the pear genome [25]. The gene *PbUGT72AJ2* was identified by bioinformatics analysis and RT-qPCR, which is considered to be a monolignol glycosylation-related *UGT*. Through the evolutionary tree analysis of *PbUGT72AJ2* and *UGTs* that have been identified as functional in other species, it was found that *PbUGT72AJ2* was clustered together with *PtGT1* [35], *AtUGT72E1*, *AtUGT72E2*, *AtUGT72E3* [16], *CsUGT72AM1* [36], *AtUGT72B1*, *AtUGT72B2*, *AtUGT72B3* [33], and *P. tremula x P. alba UGT72B37* (*PtUGT72B37*) [37], as shown in Figure 1. In addition, the alignment analysis indicated that *PbUGT72AJ2* had 55.08%, 50.72%, and 40.61% identity from *Populus tomentosa PtGT1* (GenBank: HM776516) [35], *Camellia sinensis CsUGT72AM1* (GenBank: KY399734) [36] and *P. tremula x P. alba UGT72B37* (*PtUGT72B37*) [37], respectively. The sequence alignment results showed a conserved PSPG box which is related to the binding of different types of sugar donors in the C-terminus of *PbUGT72AJ2* (Figure 2).

### 2.2. In Situ Hybridization Showed That Most PbUGT72AJ2 Transcripts Were Distributed in Lignin Deposition and Stone Cell Areas of Pear Fruit

**Figure 3:**
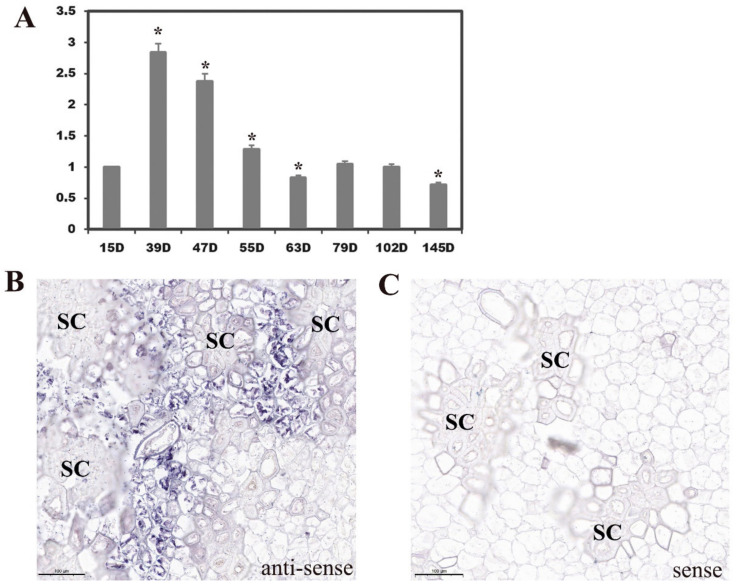
RT-qPCR analysis of *PbUGT72AJ2* in pear fruits different developmental stages and RNA in situ hybridization illustrating *PbUGT72AJ2* transcript localization at 39 days after flowering (DAF) in pear fruit. The relative expression of *PbUGT72AJ2* in different developmental stages of pear fruit (**A**). The value on the left Y-axis indicates the relative gene expression levels; error bars represent the standard (SE) of three biological replicates; * *p* < 0.05. Sections were taken through the flesh and probed with anti-sense (**B**) and sense probes (**C**) and imaged at 10 magnification. D: days after flowering; the scale bar = 100 μm; SC: stone cells.

We first executed RT–qPCR analysis of *PbUGT72AJ2* in pear fruits at different developmental stages. The results indicated that the expression level of *PbUGT72AJ2* was high in the early stage and low in the later stage of pear fruit development, showing an overall trend of rising firstly and then declining. Among them, *PbUGT72AJ2* had the highest expression level in the stage of 39 DAF (days after flowering) (Figure 3A). To verify that *PbUGT72AJ2* gene expression is associated with those cells undergoing lignin deposition and secondary wall thickening, we carried out RNA in situ hybridization [39] on fresh pear fruit 39 days after flowering. The positive expression of BCIP/NBT was blue–purple. The results of *PbUGT72AJ2* transcripts via in situ hybridization with the *PbUGT72AJ2* anti-sense probe demonstrated that *PbUGT72AJ2* transcripts were localized to some pulp cell walls, lignin deposition and stone cell areas of pear fruit (Figure 3B). Hybridization with the control *PbUGT72AJ2* sense probe showed that no hybridization was detected in the pulp cell walls, stone cells or other areas of pear fruits. (Figure 3C).

### 2.3. Enzymatic Assays of Recombinant PbUGT72AJ2 In Vitro

**Figure 4:**
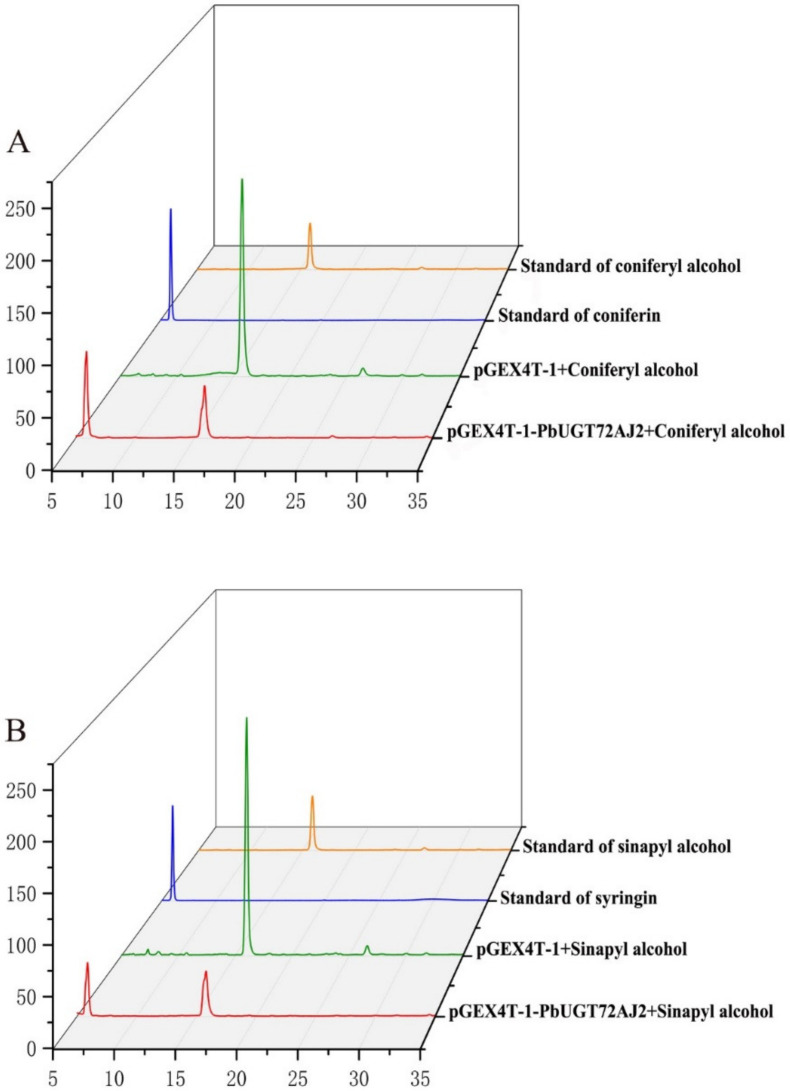
Enzyme activity of PbUGT72-pGEX-4T-1 recombinant protein toward coniferyl alcohol and sinapyl alcohol. (**A**) HPLC analysis of Standards: coniferyl alcohol, coniferin, and after reaction coniferyl alcohol with PbUGT72AJ2-pGEX-4T-1 recombinant protein. (**B**) HPLC analysis of Standards: sinapyl alcohol, syringin, and after reaction sinapyl alcohol with PbUGT72AJ2-pGEX-4T-1 recombinant protein.

*PbUGT72AJ2* was cloned into the pGEX4T-1 vector, and then, the constructed vector was induced and expressed in BL21(ED3) *E. coli*. A target protein with a weight of 78.7 kDa was obtained (GST: 26 kDa; PbUGT72AJ2: 52.7 kDa). Then, sodium dodecyl sulphate–polyacrylamide gel electrophoresis (SDS–PAGE) analysis and verification were performed (Figure S1). This was done to verify whether the recombinant protein has the ability to glycosylate monolignols of pear lignin, coniferyl alcohol and sinapyl alcohol. We added recombinant protein, coniferyl alcohol, sinapyl alcohol, uridine diphosphate glucose (UGT–glucose), and buffer in a certain reaction system. After the reaction was allowed to proceed for a period of time, methanol was added to terminate the reaction, and then it was submitted to HPLC for detection. Coniferyl alcohol, sinapyl alcohol, coniferin, and syringin were put into HPLC for reference (Figure 4A,B). The results showed that after the recombinant protein was added, two peaks of substrate (coniferyl alcohol and sinapyl alcohol) and product (coniferin and syringin) were detected by HPLC (Figure 4A,B); however, in the control group with GST-tagged protein, only coniferyl alcohol and sinapyl alcohol were detected, and no coniferin and syringin were found. This phenomenon indicates that the recombinant protein is active against both substrates. To calculate the kinetic parameters of the recombinant protein for coniferyl alcohol and sinapyl alcohol enzymes, 10 substrates with different concentrations were used in this study. The K_m_ V_max_ and k_cat_ values of PbUGT72AJ2-PGEX4T-1 were calculated through the double reciprocal attack diagram. The results are shown in Figures S2 and S3 and Table 1, and are compared with sinapyl alcohol; PbUGT72AJ2-PGEX4T-1 was found to have a stronger affinity for coniferyl alcohol and a faster catalytic efficiency for coniferyl alcohol.

In addition, optimization conditions of PbUGT72AJ2 were tested at different pHs and temperatures of coniferyl alcohol as the substrate (Figure S4). A pH of 4–10 was used for the reaction, and the reaction temperature tolerance ranged from 10 to 60 °C. The results showed that the optimal reaction pH was 7 in vitro, and 30 °C showed the highest catalytic activity.

### 2.4. The PbUGT72AJ2 Gene Was Transferred into Arabidopsis thaliana Mutants and Arabidopsis thaliana

**Figure 5:**
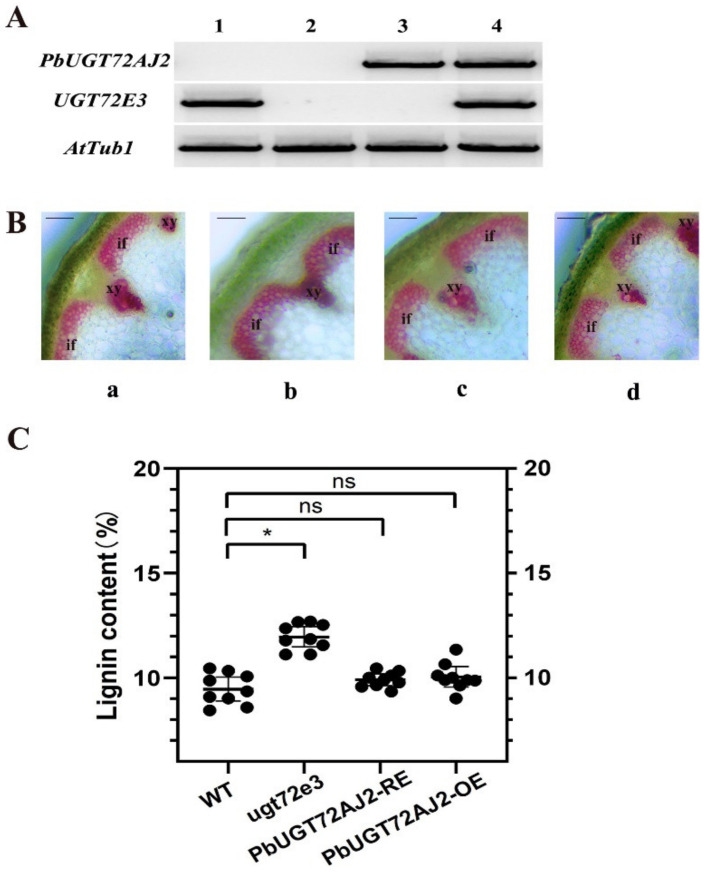
The lignin content in inflorescence stems of *Arabidopsis thaliana*. (**A**) RT-PCR analysis of *Arabidopsis thaliana* wild type (1), *UGT72E3* mutant (2), *UGT72E3* mutant lines rescued by *PbUGT72AJ2* (3) and *PbUGT72AJ2* overexpressing *Arabidopsis thaliana* (4). (**B**) cross-sections of the inflorescence stem were stained with phloroglucinol–HCl (lignin appears red when dyed); (a) Wild-type (WT) plants; (b) *UGT72E3* knockout mutant plants; (c) *PbUGT72AJ2* rescue plants; (d) over-expression *PbUGT72AJ2* plants. The bar = 100 μm; xy, xylem tracheary elements; if, interfascicular fiber cells. (**C**) Measurement of the lignin content in inflorescence stems of *Arabidopsis thaliana*. The asterisks indicate significant differences compared to the wild-type plants (* *p* < 0.05; ns, no significance).

We transferred the *PbUGT72AJ2* gene into *Arabidopsis* *thaliana* mutants (*PbUGT72AJ2-RE*) and wild-type *Arabidopsis* *thaliana* (*PbUGT72AJ2-OE*). By reverse transcription PCR (RT–PCR), we found that wild-type *Arabidopsis thaliana* only expressed *AtUGT72E3* (Figure 5A(1)), whereas neither *AtUGT72E3* nor *PbUGT72AJ2* transcripts were found in *Arabidopsis thaliana* mutants (Figure 5A(2)). The rescue lines (Figure 5A(3)) were proven to express the expected putative *PbUGT72AJ2* gene but not the *AtUGT72E3* gene; however, the overexpressed *Arabidopsis thaliana* (Figure 5A(4)) had both transcripts. To directly observe the in situ distribution of lignin in inflorescence stems of transgenic *Arabidopsis* *thaliana* and *Arabidopsis* *thaliana* mutant plants, we performed Wiesner (phloroglucinol-HCl) histochemical staining [40] of the inflorescence stems of *Arabidopsis* *thaliana* plants. The Wiesner staining results indicated that the xylem and intravascular fibres in the stems of *Arabidopsis* *thaliana ugt72e3* mutants showed stronger phloroglucinol staining compared to the wild-type plants (Figure 5B). Then, the effect of deepening lignin thickness disappeared by sectioning the rescue line *Arabidopsis thaliana* with the *PbUGT72AJ2* gene transferred into the *Arabidopsis thaliana ugt72e3* mutant. Finally, we observed stained sections of *PbUGT72AJ2-OE Arabidopsis* *thaliana* and found that lignin deposition in overexpressed *Arabidopsis thaliana* did not increase to any extent compared with that in wild-type *Arabidopsis thaliana*. Subsequently, we measured the lignin content of wild-type, *Arabidopsis* *thaliana ugt72e3* mutant, *PbUGT72AJ2-RE* and *PbUGT72AJ2-OE Arabidopsis* *thaliana* by the acetyl bromide method [41] (Figure 5C). The lignin content of the *Arabidopsis* *thaliana ugt72e3* mutant was higher than that of the wild-type, but lignin deposition was alleviated after the mutant was transferred into the *PbUGT72AJ2* gene. However, the overexpression plants did not change compared with the wild type.

In addition, the content of coniferin and syringin extracted from WT, *ugt72e3* mutant, *PbUGTAJ2-RE,* and *PbUGT72AJ2-OE Arabidopsis* *thaliana* young stems was also analyzed (Table 2). Knockout of *UGT72E3* resulted in a reduction in coniferin and syringin compared to wild-type Arabidopsis, but the reduction was not severe. This phenomenon may be caused by the functional redundancy of Arabidopsis homologous gene. After transfer into *PbUGT72AJ2*, the phenomenon of coniferin and syringin deletion has been restored to a certain extent and overexpression resulted in an almost multiple increase in the content of coniferin and syringin (Table 2).

### 2.5. Transient Transformation of PbUGT72AJ2 and PbUGT72AJ2-RNAi in Pear Fruit

**Figure 6:**
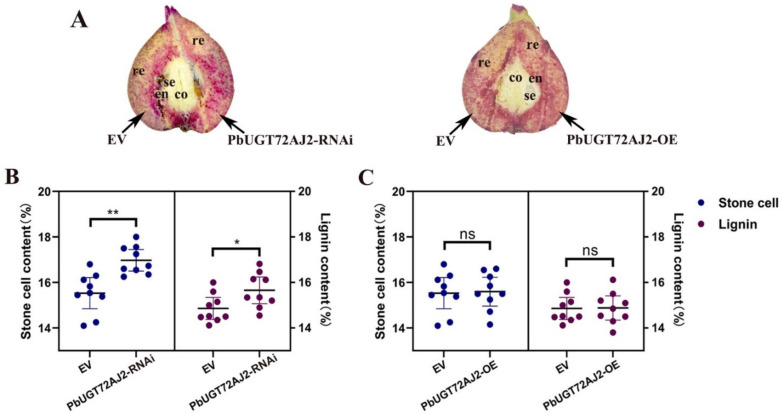
Transient assays using *PbUGT72AJ2* silencing and overexpression constructs in ‘Dangshansuli’ fruit at 39 days after flowering (DAF). Images were taken 10 days after agro-infiltration. (**A**) cross-sections of the pear fruits were stained with phloroglucinol–HCl. re, receptacle; en, endocarp; se, seed; co, core. (**B**,**C**) Lignin and stone cell contents in the flesh tissue around the infiltration sites, more than three fruits were injected with each construct in an experiment that was repeated three times. EV: empty vector; (* *p* < 0.05, ** *p* < 0.01; ns, no significance).

**Figure 7:**
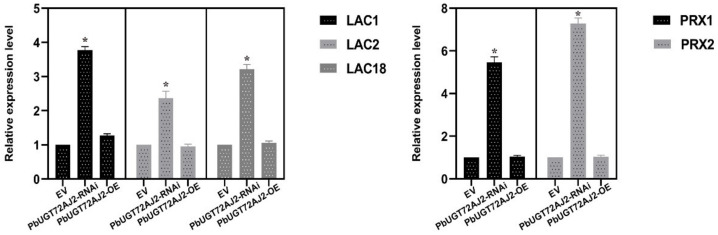
RT-qPCR confirmed the activation of genes (*LAC* and *PRX*) responsible for secondary wall biosynthesis in *PbUGT72AJ2-RNAi* and *PbUGT72AJ2-OE*. EV: empty vector. * *p* < 0.05. Error bars represent the standard (SE) of three biological replicates.

Since the stable genetic transformation system of pears is in its infancy, it is difficult to perform functional analysis of *PbUGT72AJ2* in pears. Therefore, we chose the transient injection method for evaluation. We transiently injected the *PbUGT72AJ2* gene and RNAi silenced *PbUGT72AJ2* in the 39 DAF (days after flowering) fruits of pear tree. By Wiesner (phloroglucinol-HCl) histochemical staining [40], we found that when *PbUGT72AJ2* was overexpressed, the change in pear stone cells was not obvious, but after the *PbUGT72AJ2* gene was silenced by RNAi intervention, it was found that the pear stone cells increased compared with the empty vector injection in the control (Figure 6A). The current study statistically analysed the contents of stone cells and lignin in pear fruit. The contents of stone cells and lignin in pear fruit were statistically analysed. These results are shown in Figure 6B,C. When *PbUGT72AJ2* was silenced, the contents of stone cells and lignin in pear fruit were increased compared with empty vector injection (Figure 6B). However, the contents of stone cells and lignin in pear fruit overexpressing *PbUGT72AJ2* after transient injection did not change compared with empty vector injection (Figure 6C). Subsequently, we extracted RNA from control, *PbUGT72AJ2-OE,* and *PbUGT72AJ2-RNAi* pear fruits and performed fluorescence quantitative analysis of *PbUGT72AJ2* downstream laccase and peroxidase genes (Figure 7). The laccase and peroxidase functional genes and their gene number are from Xue et al. [42]. The results showed that compared with the control group, the expression levels of *LAC1*, *LAC2*, *LAC18*, *PRX1,* and *PRX2* were significantly increased in the RNA interference pear fruit, whereas the expression levels of *LAC1*, *LAC2*, *LAC18*, *PRX1,* and *PRX2* did not change in the *PbUGT72AJ2* overexpressed pear fruit.

## 3. Discussion

Several recent publications have described the potential role of glucosylation in phenylpropanoid homeostasis in plants [43,44,45]. In recent years, as our research on lignin has progressed, researchers have begun to examine the regulation and transportation mechanisms of lignin, as well as the role of important genes in the lignin pathway. Uridine diphosphate glycosyltransferase (UGT), as an important transport gene in the pathway of lignin formation, has received extensive attention. In recent studies, it was found by transcriptome analysis that the pomegranate soft-seeded cultivar is due to a large amount of lignin monolignols in the inner seed coat, and it is conjugated into monolignol glucosides and stored. The monolignols can also be transported to the cell wall and used as lignin precursors, resulting in a hard-inner seed coat phenotype. [46]. The amounts of glysosylated phenolic compounds in outer stem tissues containing fibres with very low amounts of cell wall lignins and lignin-rich xylem tissues have been described as having an inverse correlation in a comparative study of flax [47].

In *Arabidopsis* *thaliana*, a cluster of glucosyltransferases, including *UGT72E1*, *UGT72E2* and *UGT72E3*, was identified to be responsible for the glucose conjugation of monolignols [16,29]. To explore whether PbUGT72AJ2 can catalyse lignin monolignols in the lignin formation pathway, we conducted a substrate enzyme activity catalysis experiment and found that the PbUGT72AJ2-PGEX4T-1 recombinant protein can catalyse the transformation of lignin monolignols to lignin monolignol glycosides in pear. Earlier studies on the *Arabidopsis thaliana UGT72E* family showed that *UGT72E2* overexpression resulted in a large accumulation of lignin monolignols in roots and leaves [31]. Moreover, *UGT72E3* overexpression in leaves is highly similar to *UGT72E2* overexpression. They both lead to the production of massive levels of coniferyl and sinapyl alcohol 4-O-glucosides and a substantial loss in sinapoyl malate [32]. Recent studies have also found that the knockout of *AtUGT72E3* can lead to increased lignin deposition. The author proposed that knockout of the *AtUGT72E3* gene leads to increased monolignol flow to the cell wall and is accompanied by an increase in the expression of downstream peroxidase *PRX* and laccase *LAC* [34]. To verify whether the increase in downstream gene expression was caused by the knockout of *PbUGT72AJ2*, we extracted the silenced *PbUGT72AJ2* gene 39 days after flowering pear fruit RNA. The qRT–PCR results showed that transient injection of pear fruit increased the expression of *PbLAC* and *PbPRX* genes [42] significantly after *PbUGT72AJ2* silencing (Figure 7). This phenomenon showed that the content of lignin and stone cells after *PbUGT72AJ2* silencing may be caused by the increase in downstream gene expression.

In *Arabidopsis* *thaliana*, the *ugt72b1* mutant was characterized by ectopic lignification in areas around pith tissues and interfascicular fibres, accompanied by the phenomenon of early flowering. The study highlights an important role of monolignol glycosylation in maintaining the homeostasis of the monolignol biosynthesis pathway and lignin deposition by investigation of *ugt72b1* mutants [33]. Competition between monolignol glucosylation and lignin formation may exist. When *UGT72B1* works normally, monolignol glucosylation catalysed by *UGT72B1* maintains a balance between monolignols and their glucosides. Although the overexpression of *UGT72B1* will lead to an increase in glycoside forms of certain lignin monolignol glycosides, the increased part may be stored in vacuoles and will not lead to substantial changes in lignin content, and *UGT72B1* knockout will also lead to significant up-regulation of some genes in the lignin pathway [33]. However, a study that overexpressed poplar *PtGT1* in tobacco obtained the opposite results. Wiesner and Maule staining of overexpressed *PtGT1* tobacco showed that the stem xylem of transgenic tobacco plants stained more strongly compared to controls, and the transgenic lines showed much higher lignin content than control plants by measurement of Klason lignins [35]. Furthermore, the ectopic overexpression of *PtGT1* in tobacco resulted in an early flowering phenotype. However, the authors believe that the role of *PtGT1* in lignin biosynthesis was not directly related to the glycosylation of lignin monolignols because the PtGT1 recombinant protein does not recognize lignin monolignols as a substrate, and transgenic tobacco plants overexpressing *PtGT1* do not contain higher levels of lignin monolignol glycosides. Therefore, further studies are needed to better understand these molecular pathways. [35].

In our experiment, through the observation of the *Arabidopsis* *thaliana ugt72e3* mutant, *PbUGT72AJ2-OE* and *PbUGT72AJ2-RE* Wiesner (phloroglucinol-HCl), after histochemical staining of inflorescence stems and the lignin content of *UGT72E3* mutants *PbUGT72AJ2-OE* and *PbUGT72AJ2-RE* as measured by the acetyl bromide method [40,41], we found that the lignin content of the *Arabidopsis* *thaliana ugt72e3* mutant was higher than that of the wild-type, but the lignin deposition was alleviated after the mutant was transferred into the *PbUGT72AJ2* gene. However, the overexpression in plants did not seem to change compared with the wild-type. To verify the effect of *PbUGT72AJ2* on the *Arabidopsis* *thaliana* monolignol glucoside content, we also measured the monolignol glycoside content of wild-type, *ugt72e3* mutant, *PbUGT72AJ2-OE,* and *PbUGT72AJ2-RE Arabidopsis* *thaliana* inflorescence stems (Table 2). The results showed that compared with the wild-type, the *ugt72e3* mutant coniferin and syringin content seemed to not decrease, which may be the reason for the functional redundancy of the *UGT* gene. In contrast, overexpression of the *PbUGT72AJ2* gene resulted in an increase in these two glycosides, but it did not lead to an increase in lignin. This phenomenon also verified the theory that most of the lignin monolignols turned into glycosides and may be stored in vacuoles [48,49,50]. To further explore whether *PbUGT72AJ2* can further regulate the development of stone cells by regulating the production of lignin, we carried out in situ hybridization and transient transformation experiments. In situ hybridization showed that the transcripts of *PbUGT72AJ2* were localized to some pulp cell walls, lignin deposits and stone cell areas of pear fruit. When *PbUGT72AJ2* was transiently expressed in pear fruit, the stone cells and lignin of 39 DAF (days after flowering) pear fruit did not change significantly, but when the gene was silenced in pear fruit, the lignin and stone cells of 39 DAF (days after flowering) pear fruit increased to a certain extent. 

## 4. Materials and Methods

### 4.1. Plant Materials, Treatments, and Growth Conditions

The annual fruit samples were sampled from 30-year-old pear trees (*P. bretschneideri*) that were grown in a horticultural field (Dangshan, Anhui, China). We collected the fruit on April 15th (15 days after flowering), May 14th (39 DAF), May 22nd (47 DAF), June 4th (55 DAF), June 12th (63 DAF), June 28th (79 DAF), and August 30th (145 DAF). Each sample was stored at −80 °C for subsequent RNA extraction. 

*Arabidopsis thaliana* L. wild type (Col-0 ecotype) was purchased from the American *Arabidopsis* *thaliana* Biological Resources Center. *Arabidopsis* *thaliana At5g26310* (*UGT72E3*, Col-0 ecotype) mutant seeds corresponding to the SAIL_1279_D02 flanking sequence tag were ordered from the Salk Institute Genomic Analysis Laboratory.

The *Arabidopsis* *thaliana* plants were cultivated in a growth chamber at 23 °C under 16/8 h light/dark cycles (constant illumination 100 μE m^−2^s^−1^). Ten replicates of each line were planted, and three similarly growing plants were collected for further analysis.

### 4.2. RNA Extraction, Reverse Transcription PCR and RT–qPCR Analysis

Extraction of total RNA from each frozen tissue was performed using an RNAiso-mate for the Plant Tissue kit (Tiangen, Beijing, China). Total RNA (1 µg) from each sample was used in reverse transcription. First-strand cDNA was synthesized with a PrimeScript™ RT reagent kit with a gDNA Eraser kit (TaKaRa, Kyoto, Japan). *Arabidopsis* *thaliana* plant identification primers are listed in Table S1.

All RT–qPCR primers were designed using Primer Premier 5.0 software (Premier, Ottawa, ON, Canada) (Table S2). We chose tubulin (No. AB239680.1) as the reference gene for RT–qPCR analysis [51]. RT–qPCR was performed with a CFX96 Touch™ Real-Time PCR Detection System (BIO-RAD, Louisville, KY, USA). The relative changes in gene expression levels were calculated using the 2^−∆∆CT^ method [52].

### 4.3. Sequence Alignment and Phylogenetic Analysis

In this study, the amino acid sequence alignment analysis of *UGTs* was conducted using DNAMAN 7.0 software (LynnonBiosoft, San Ramon, CA, USA). A phylogenetic analysis using the amino acid sequences of *UGT* members was performed using MEGA 7.0 software [38] and a phylogenetic tree was constructed using neighbor-joining distance analysis. The tree nodes were evaluated with the bootstrap method for 1000 replicates, and the evolutionary distances were computed using the *p*-distance method [38].

### 4.4. In Situ Hybridization

Segments of fresh pear fruit at 39 DAF were fixed in in situ hybridization fixation solution overnight. Organization fixed: The organization was removed, washed clean, and then immediately placed in the fixed fluid (DEPC) to fix for more than 12 h. Dehydration: The tissue was dehydrated by gradient alcohol, paraffin, embedding, and vacuum pumping in a dehydration process. Section: The paraffin was sliced using a slicer, made into a piece using the slice machine and then placed in a 62 °C oven for roasting for 2 h. Dewaxing and dehydration: Sections were soaked in 2 changes of xylene for 15 min each. The samples were dehydrated in 2 changes of pure ethanol for 5 min each [39]. Then, the samples were dehydrated in gradient ethanol solutions of 85% and 75% ethanol for 5 min each. They were washed in DEPC dilution. Digestion: We marked the objective tissue with a liquid blocker pen, according to the characteristics of tissues, added proteinase K (20 µg/mL) working solution to cover the objectives, and incubated at 37 °C for 22 min. They were washed with pure water, and then washed three times with PBS (pH 7.4) in a rocker device for 5 min each. Hybridization: We discarded the prehybridization solution (Servicebio, Wuhan, China), added the probe hybridization solution (Servicebio, Wuhan, China), concentrated and incubated the section in a humidity chamber, and hybridized overnight. Developing NBT/BCIP: the sections were dried slightly, and we added freshly prepared NBT/BCIP chromogenic reagent to the marked tissue [39]. We observed the reaction time under a microscope until positive expression appeared blue–purple. Then, the developing reaction was stopped by washing in running tap water [39]. The probe sequence was unique to the *PbUGT72AJ2* locus (Table S1) and resulted in a single hit when used as a quarry to BLAST the Chinese white pear genome.

### 4.5. Genetic Transformation of Arabidopsis thaliana and Transient Transformation of Pear Fruit

The full-length coding sequence of *PbUGT72AJ2* was cloned from first-strand complementary DNA (cDNA) with KOD OneTM PCR Master Mix-Blue (TOYOBO, Osaka, Japan). The fragment was cloned into pCAMBIA1301-35S binary vectors and used in plant transformation. Wild-type (Col-0 ecotype) and *At5g26310* (*UGT72E3*, Col-0 ecotype) mutants were used for transformation with Agrobacterium tumefaciens GV3101 carrying the above binary plasmid using the floral dip method.

The full-length coding fragment was cloned into pCAMBIA1301-35S binary vectors and used in transient injection of pear fruit overexpression. Hairpin constructs were based on the pRNAiDE001 vector [53,54], where self-complementary sense (431-725bP) and antisense sequences are separated by a nonfunctional sequence (loop), and then this fragment was cloned into the pCAMBIA1301 vector with the 35S promoter. For transient injection, 30-year-old pear trees and young fruits 39 days after flowering were selected. The constructed pcambina1301-35s binary vectors of *PbUGT72AJ2* overexpression and the *PbUGT72AJ2* RNA interference gene were injected on the left of the fruit, and the empty pcambina1301-35s binary vectors were injected on the right for control. The method followed a previously reported protocol, and nine fruits were injected with each construct in an experiment that was repeated three times independently. The method followed a previously reported protocol [55]. After 10 days, the transiently injected fruits were picked up.

### 4.6. Construction of the PbUGT72AJ2 Expression Vector and Induced Purification of Recombinant Protein

The full-length coding sequence of *PbUGT72AJ2* was cloned and then expressed in the pGEX4T-1 vector (GE Healthcare, Chicago, IL, USA). Recombinant pGEX4T-1-PbUGT72AJ2 was transformed into *E. coli* BL21 (DE3)-competent cells. The culture was expanded to an OD value of approximately 0.6, and isopropyl-β-d-thioacetamide IPTG was added to a final concentration of 1 mM/L to induce the fusion expression of the target protein and GST binding protein. Finally, the cells were cultured with shaking at 16 °C for 24 h, and the cells were collected by centrifugation at 4 °C. Affinity chromatography resin with GST tag GST • Bind™ resin (Novagen, Darmstadt, Germany) was used to purify the protein, and elution buffer (pH 8.0) was used for GST-Sefinose (TM) resin (containing reducing glutathione reagent at a concentration of 1.538 g/L) elution.

### 4.7. PbUGT72AJ2-GST Recombinant Protein Enzyme Assays 

To analyse the activity of the recombinant protein PbUGT72AJ2-GST, 10 μg of purified protein was incubated at 35 °C with 20 μL buffer (50 mM MgSO4, 200 mM KCl, 100 mM PBS pH 7.2–7.4), 2.5 mM UDP-glucose, and 1 mM substrates (coniferyl alcohol and sinapyl alcohol). Water was added to a final volume of 50 μL, and after 1 h of reaction, 50 μL methanol termination reaction was added. All reactions were supplemented with 0.1% (*v*/*v*) β-mercaptoethanol. Then, the reaction results were analysed by HPLC. 

To calculate the K_m_, V_max,_ and K_cat_ values of the recombinant protein, under the same original reaction conditions, the substrates (coniferyl alcohol and sinapyl alcohol) were set to 10 concentrations (0.025 mm, 0.05 mm, 0.075 mm, 0.1 mm, 0.15 mm, 0.2 mm, 0.3 mm, 0.4 mm, 0.5 mm, and 0.6 mm). Then, the product concentration C was calculated by the external standard method, and the reaction rate V was further calculated [36].

### 4.8. Lignin Determination and Histochemical Staining of Arabidopsis thaliana and Pear Fruits

We collected *Arabidopsis* *thaliana* plants for approximately 6 weeks, removed the leaves, and dried them in an oven at 65 °C for 48 h. The lignin content of the *Arabidopsis thaliana* plants was estimated following the method of Anderson et al. [41]

We obtained the inflorescence stem segments (young stem regions) of the transgenic and wild-type *Arabidopsis* *thaliana* plants for approximately 6 weeks. The samples were placed in a solution containing 95% methanol, 70% (*v*/*v*) ethanol, and glacial acetic acid for 12 h and they were embedded in paraffin for sectioning with a pathology slicer (RM 2018). Plant tissue sections were stained following standard phloroglucinol staining protocols [40].

After the transiently injected pear fruit was brought back to the laboratory, 5 g of pulp was sampled from 2.0 mm under the peel to 0.5 mm outside the core, collected, and frozen at −80 °C for 24 h, and homogenized with a high-speed homogenizer for 3 min at a rotating speed of 20,000 rpm/min; water was added and the fruit stood for a while. Then, the upper suspension was poured out, and this was repeated several times until the upper layer was clear, and filtered; the dried stone cells were weighed, this was repeated 3 times, and stone cell content = measured stone cell dry weight/5 × 100%. The lignin content was measured using the Klason method [56]. A small amount (0.2 g) of stone cells was extracted with 15 mL of 72% H_2_SO_4_ at 30 °C for 1 h, combined with 115 mL of distilled water, and boiled for 1 h. The volume was kept constant during boiling. The liquid mixture was filtered and the residue was rinsed with 500 mL of hot water, air-dried, and weighed. The lignin content was shown as a percentage (calculated lignin content/dry weight of stone cells × 100%) [55].

### 4.9. Targeted Metabolite Determination

HPLC (Thermo Scientific UltiMate 3000 HPLC, Waltham, MA, USA) analysis was carried out using a Columbus (Thermo Scientific 5-μm C18 column; 250 × 4.60 mm). Acetonitrile (solvent A) and H_2_O (solvent B) with a gradient of 10–30% acetonitrile in water (all solutions contained 0.1% trifluoroacetic acid) with a flow rate of 1 mL min^−1^ over 35 min were used [33]. Each peak on the chromatogram was scanned between 200 and 400 nm (photodiode array profile) and was integrated at 264, 280, and 324 nm. The data was acquired and analysed using ChromQuest version software (Waltham, MA, USA). Tissue extraction was carried out as described in Lanot et al. [31].

## 5. Conclusions

Lignin deposition in pear fruit is closely related to stone cell development and glycosyltransferase-mediated transport of monolignols is essential for the formation of lignin. In our previous work, we identified a gene, *PbUGT72AJ2*, which may be involved in lignin monolignol glycosylation. In this study, we investigated the localization of *PbUGT72A2* transcripts in pear fruits and analysed the enzymatic kinetics of PbUGT72AJ2. We verified the effects of *PbUGT72AJ2* transferred into *Arabidopsis thaliana* and *Arabidopsis thaliana* mutants, as well as the effects of transient overexpression and interference on pear fruit stone cell development and lignin formation. In conclusion, a series of experiments proved that *PbUGT72AJ2* mediated glycosylation by catalyzing the glucose conjugation of monolignols and may affect the expression of downstream genes as well as the content of monolignols to affect the lignin deposition and stone cell development in pear fruit.

## Figures and Tables

**Table 1:** Enzyme kinetics of PbUGT72AJ2.

Substrates	K_m_ (μM)	V_max_ (μM/min·mg)	K_cat_ (S^−1^)	K_cat_/K_m_ (mM^−1^S^−1^)
Coniferyl alcohol	117.83 ± 7.94	4.23 ± 0.15	0.55 ± 0.02	4.67 ± 0.13
Sinapyl alcohol	144.13 ± 22.31	3.32 ± 0.27	0.44 ± 0.03	3.05 ± 0.25

**Table 2:** HPLC Analysis of soluble monolignol glucosides metabolites in *Arabidopsis*.

Materials	Genotypes	Coniferin μmol/5 g	Syringin μmol/5 g
Inflorescence stems	WT	0.112 ± 0.02	0.067 ± 0.02
	UGT72E3-KO	0.093 ± 0.01	0.055 ± 0.02
	PbUGT72AJ2-RE	0.103 ± 0.01	0.062 ± 0.01
	PbUGT72AJ2-OE	0.213 ± 0.04	0.102 ± 0.03

## Data Availability

The PbUGT72AJ2 gene and amino acid sequence of pears used in this experiment can be obtained from NCBI (GenBank: KR270486).

## Supplementary Materials

The following supporting information can be downloaded at: https://www.mdpi.com/article/10.3390/ijms23147893/s1.

## Abbreviations

ABC transporter: ATP-binding cassette transporter. pCAG: *p*-coumarin glycosides; qRT-PCR: Real-Time PCR; EV: empty vector; RE: Rescue expression; OE: Over expression; PRX: POD, Peroxidase; LAC: Laccase.
