# Supplementary material for: PbUGT72AJ2-Mediated Glycosylation Plays an Important Role in Lignin Formation and Stone Cell Development in Pears (Pyrus bretschneideri)
Source: Int J Mol Sci. 2022 Jul 18;23(14):7893. doi: 10.3390/ijms23147893 (PMC9318811; doi:10.3390/ijms23147893)
Supplement: Supplementary file 1 [file ijms-23-07893-s001.zip › Supplementary information/Table S1.pdf]

Table S1. Sequence of primers for gene cloning.

Gene name	Primer sequences 5'	Primer sequences 3'
PbUGT72AJ2	ATGAGCTCAAAGCCACATGCTG	CTAGCATTTTCTTTGATTGCCAC
pGEX4T-1-PbUGT72AJ2	TCCCCCGGGATGAGCTCAAAGCCACATGCTG	AAGGAAAAAAGCGGCCGCCTAGCATTTTCTTTGATTGCCAC
pCambia1301-PbUGT72AJ2	TCCCCCGGGATGAGCTCAAAGCCACATGCTG	CGCGGATCCCTAGCATTTTCTTTGATTGCCAC
PbUGT72AJ2-RNAi	GTAGCTTTGACCGTCTACGTGCCA	ATGGGATAAATTGGCACCTTAGCC
PbUGT72AJ2-probe	Sense: ATTTTTTGACATTATTGAACTCCCACCGCCCGA	Anti-sense: TCGGGCGGTGGGAGTTCAATAATGTCAAAAAAT
AtUGT72E3	ATGCATATCACAAAACCACACGCC	CTAAGCACCACGTCCCAAGTCCCC
AtTub1	ATGAGAGAAATCCTCCACGTCCAA	TCAAGATTCGTAAACTTGTTCTTC
