# Supplementary material for: PbUGT72AJ2-Mediated Glycosylation Plays an Important Role in Lignin Formation and Stone Cell Development in Pears (Pyrus bretschneideri)
Source: Int J Mol Sci. 2022 Jul 18;23(14):7893. doi: 10.3390/ijms23147893 (PMC9318811; doi:10.3390/ijms23147893)
Supplement: Supplementary file 1 [file ijms-23-07893-s001.zip › Supplementary information/Table S2.pdf]

Table S2. Sequence of primers for qRT-PCR.

Gene name	Primer sequences 5’	Primer sequences 3’
PbUGT72AJ2	GTTGACAGAGAAGAGATA	TTATATGATGAACCACCTA
PbLAC1	GTAGCTATCCGATTTATCGCA	AGAGATTGATTAGGTCCTTTGC
PbLAC2	CTTACCTTCTACGAATCATC	GGCATCTACTTCCACTAT
PbLAC18	CACTCCACCTAACAACAC	CAACACCAATTCCACACTA
PbPRX1	GGTTTGATGATGGTGGAT	AAGTAGTCTTGGCTCTTG
PbPRX2	ACTTCAGCACTAATCAGA	CGAATCTCTCCATCAGTA
Tubulin	AGAACAAGAACTCGTCCTAC	GAACTGCTCGCTCACTCTCC
