# Supplementary figures and images for: PbUGT72AJ2-Mediated Glycosylation Plays an Important Role in Lignin Formation and Stone Cell Development in Pears (Pyrus bretschneideri)
Source: Int J Mol Sci. 2022 Jul 18;23(14):7893. doi: 10.3390/ijms23147893 (PMC9318811; doi:10.3390/ijms23147893)

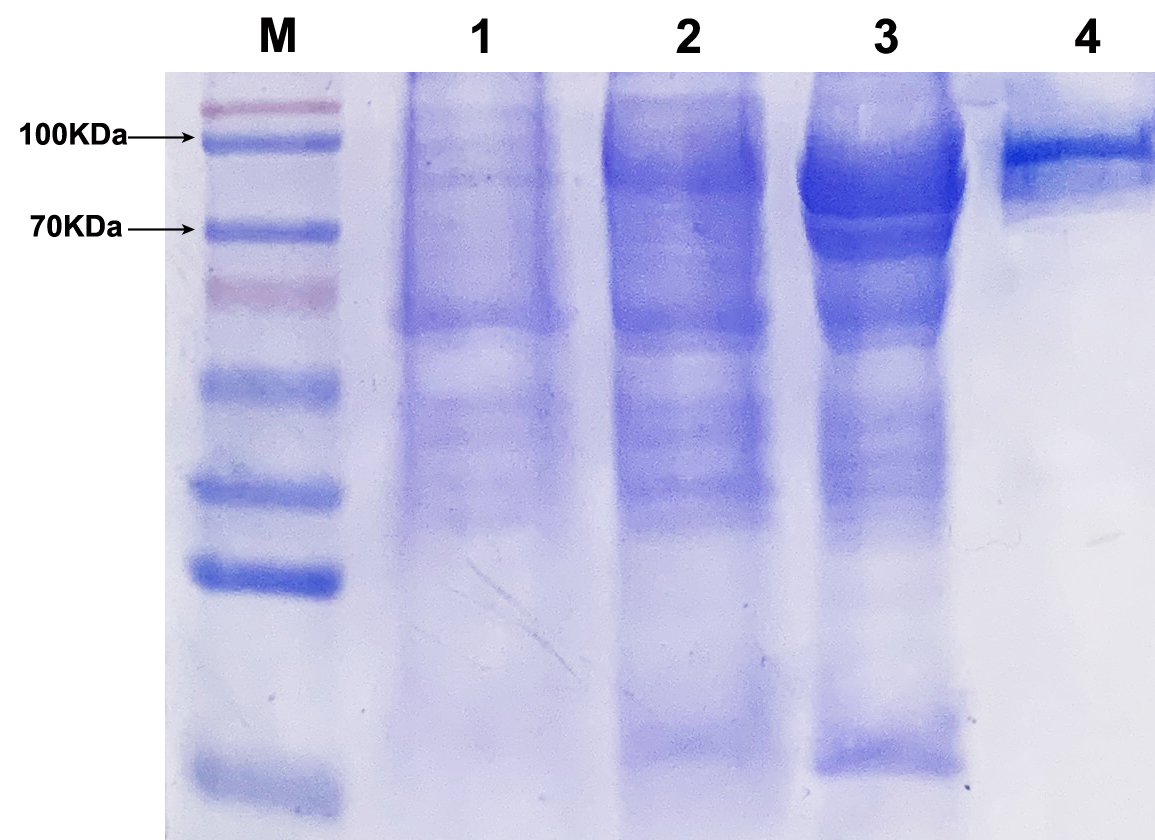

Supplement: Supplementary file 1 [file ijms-23-07893-s001.zip › Supplementary information/Figure. S1.jpg]

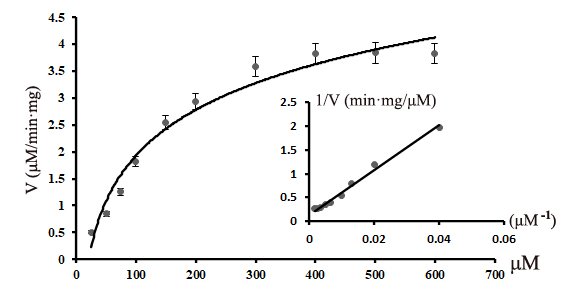

Supplement: Supplementary file 1 [file ijms-23-07893-s001.zip › Supplementary information/Figure. S2.jpg]

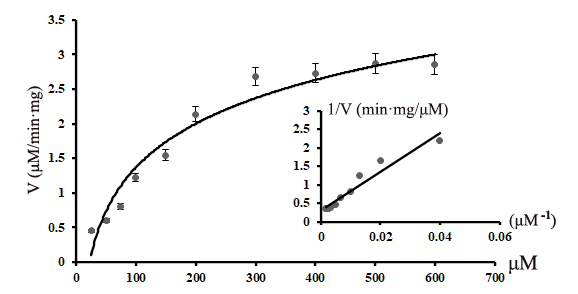

Supplement: Supplementary file 1 [file ijms-23-07893-s001.zip › Supplementary information/Figure. S3.jpg]

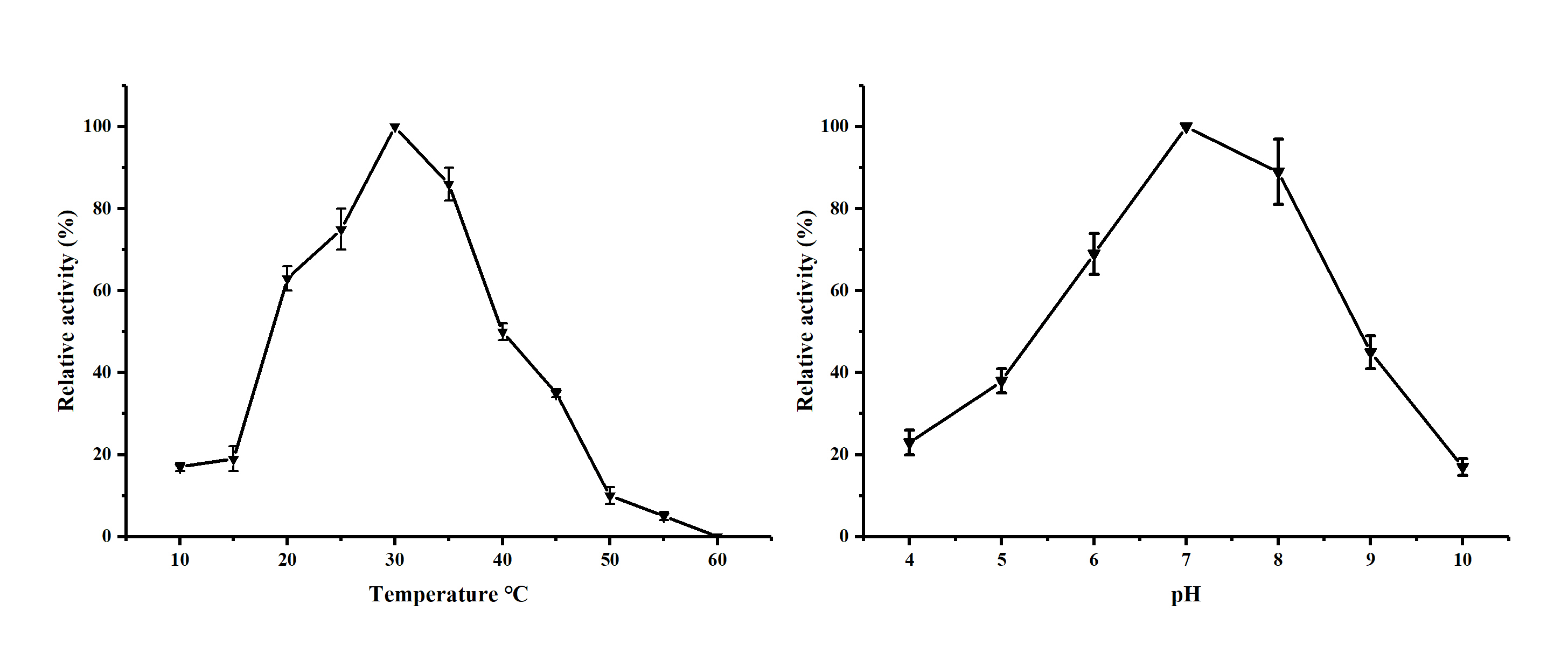

Supplement: Supplementary file 1 [file ijms-23-07893-s001.zip › Supplementary information/Figure. S4.jpg]

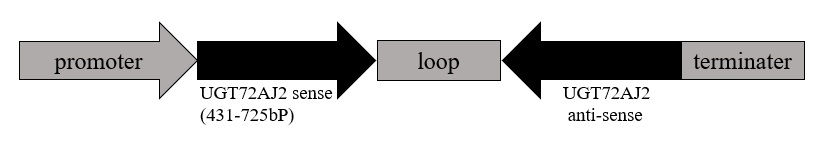

Supplement: Supplementary file 1 [file ijms-23-07893-s001.zip › Supplementary information/Figure. S5.jpg]
